# Mesenchymal stem cell-derived exosomes for the treatment of knee osteoarthritis: a systematic review and meta-analysis based on rat model

**DOI:** 10.3389/fphar.2025.1588841

**Published:** 2025-06-02

**Authors:** Zhe Wang, Zihao Hu, Lin Niu, Yongsheng Xu, Yansong Qi

**Affiliations:** ^1^ Orthopedic Center (Sports Medicine Center), Inner Mongolia Autonomous Region People’s Hospital, Hohhot, China; ^2^ Inner Mongolia Academy of Medical Sciences, Inner Mongolia Autonomous Region People’s Hospital, Hohhot, China

**Keywords:** exosomes, mesenchymal stem cells, cartilage, osteoarthritis, cartilage injury

## Abstract

**Background:**

In this study, we systematically evaluated the efficacy of mesenchymal stem cell (MSC) derived exosomes (MSC-exos) in the treatment of osteoarthritis (OA) through multimodal evaluation of cartilage protection, anti-inflammatory activity and tissue regeneration. A comparative analysis of drug delivery strategies was performed to explore better therapeutic effects.

**Methods:**

The study employed a systematic search of PubMed, Embase, and Web of Science databases for comparative studies on exosome treatments in rat knee OA models with cartilage damage up to July 2024. Two researchers independently reviewed the literature, extracted data, evaluated bias, and conducted a meta-analysis using RevMan 5.4.1, Stata IC 15, and Stata 18.

**Results:**

Our systematic review incorporated 28 preclinical studies demonstrating that MSC-exos consistently exhibited therapeutic advantages in cartilage repair, as evidenced by significant improvements across validated histological scoring systems, such as Osteoarthritis Research Society International (OARSI), Mankin, and International Cartilage Repair Society (ICRS) metrics. Mechanistic analyses revealed coordinated anabolic-catabolic modulation, with marked upregulation of cartilage-specific anabolic factors including collagen type II, aggrecan core protein, and interleukin-10. Concomitantly, MSC-exos suppressed pro-inflammatory mediators through downregulation of interleukin-1β, interleukin-6, matrix metalloproteinase-13, and tumor necrosis factor-alpha, critical regulators of extracellular matrix degradation in OA pathogenesis. Subgroup analysis of MSC types may suggest that exosomes derived from synovial fluid mesenchymal stem cells (SF-MSC-exos) and umbilical cord mesenchymal stem cells (UMSC-exos) have better effects on cartilage repair. Biweekly exosome injections are more effective than weekly injections in repairing OA.

**Conclusion:**

This meta-analysis, by combining existing evidence with network meta-analysis, suggests that UMSC-exos and SF-MSC-exos are the most effective treatment options and that twice-weekly doses are the optimal frequency of treatment. MSC-exos significantly improved the histopathological score of oOA through bidirectional regulation of cartilage anabolic activation and catabolic inhibition. The results of the subgroup analysis provide suggestions for future clinical treatment of OA with exosomes. In the future, more high-quality randomised controlled animal and clinical trials are needed to determine the optimal type, frequency and dose of exosomes for OA treatment.

**Systematic Review Registration:**

https://www.crd.york.ac.uk/PROSPERO/view/CRD42024599998, PROSPERO, CRD42024599998.

## 1 Introduction

Osteoarthritis (OA) is a degenerative disease that predominantly affects the elderly population. It is also a prevalent disabling disease, imposing a significant economic burden on both individuals and society (Martel-Pelletier et al., 2016; Yue and Berman, 2022). The underlying pathological changes are local cartilage damage, but there is also deterioration of the entire joint structure, including the synovium, ligaments, subchondral bone, articular cartilage, and periarticular muscles (Abramoff and Caldera, 2020; Yunus, Nordin, and Kamal, 2020). It is estimated that 595 million people worldwide, representing 7.6% of the global population, are affected by OA. The knee is the most commonly affected joint, with 365 million individuals experiencing symptoms (Collaborators, 2023; Cui et al., 2020). As the global population continues to age and rates of obesity and injury rise, the prevalence of OA is expected to increase.

The precise etiology of OA remains unclear. The most commonly employed therapeutic modalities for OA encompass pharmacological interventions, partial knee replacement, and artificial knee replacement, among others. Pharmacological intervention can only provide temporary symptomatic relief in patients with OA (Crawford, Miller, and Block, 2013). Joint replacement is a relatively efficacious treatment for patients with middle and advanced OA in clinical practice. However, it has several limitations, including high cost, prosthesis lifespan, and surgical risk (DeFrance and Scuderi, 2023). It is therefore a topic worthy of further study to identify safer and more effective treatments for OA. In recent years, stem cell therapy has emerged as a promising avenue for preclinical and clinical palliative care in the context of OA. Stem cells remain constrained by several limitations, including teratogenic and carcinogenic risks, immune rejection, restricted capacity for expansion *in vitro*, and ethical concerns (Derks and van Boxtel, 2023; Wuputra et al., 2020). These constraints impede further research and clinical translation. The existing literature indicates that the therapeutic effect of stem cells is primarily mediated by a paracrine mechanism, which is in turn largely dependent on exosomes (Tan et al., 2024; Lotfy, AboQuella, and Wang, 2023; Y; Sun et al., 2021). Consequently, exosomes derived from mesenchymal stem cells (MSCs) represent a promising avenue for cell-free therapy in the treatment of OA.

Exosomes are extracellular vesicles (EVs) secreted by cells that carry a variety of cellular components, including the cellular components that secrete them (DNA, RNA, lipids, metabolites, and cytoplasm and cell surface proteins). Exosomes are involved in a number of important biological processes, including angiogenesis, apoptosis, antigen presentation, intercellular signaling, and inflammation (Kalluri and LeBleu, 2020). They regulate receptor cells through intercellular communication, functioning as vital communication carriers between cells. Following endocytosis, exosomes transfer their cellular components, including proteins, lipids, and nucleic acids, to recipient cells, thereby inducing phenotypic changes in the latter (Mathieu et al., 2019). It has been demonstrated that exosomes can be identified in synovial fluid (SF) and that they exert a pivotal influence on the biological processes of OA chondrocytes and associated inflammatory cells (Chen et al., 2023). Exosomes in the SF of OA patients perpetuate synovial inflammation by recruiting immune cells, stimulating cells to produce IL-1β and IL-16, and stimulating M1 macrophages to release key molecules involved in the inflammatory process and cartilage degeneration (Domenis et al., 2017). This plays a pivotal role in the perpetuation of inflammation and the occurrence and development of OA. Exosomes regulate the biological function of OA cells by transferring microRNA (miRNA) and long non-coding RNA (lncRNA) (Xie et al., 2020). They facilitate aberrant calcification and cartilage degradation in OA and induce alterations in the phenotype of articular chondrocytes (ACs) in OA (Liu et al., 2022). The alleviation of pain is also a principal objective of OA therapy. Exosomes have been demonstrated to impede the transmission of signals between cartilage and nerve cells by overexpressing the relevant genes, thereby providing a potential avenue for pain relief in OA patients (K. Lu et al., 2023; Vadhan et al., 2024).

In order to ascertain the efficacy of MSCs-exos in improving cartilage injury and OA, we conducted a meta-analysis of existing experimental animal studies. Our objective was to provide evidence that would support further clinically relevant studies of exosomes in the treatment of knee joints.

## 2 Methods

This systematic review was conducted according to the guidelines for preclinical systematic reviews and meta-analyses in animal studies (Leenaars et al., 2012; Vesterinen et al., 2014) and reported in accordance with the guidelines of the Preferred Reporting Items for Systematic Reviews and Meta-Analyses (PRISMA) (Moher et al., 2009). To avoid duplication with other systematic reviews in progress, we searched the PROSPERO site for similar reviews beforehand and then registered our study (registration numbers: CRD42024599998).

### 2.1 Search strategy

Literature searches were conducted in July 2024 on three medical electronic databases (PubMed, Embase, and Web of science). The search strategies were devised with the objective of identifying pertinent preclinical studies evaluating the effect of exosomes derived from MSCs on OA. The following keywords were used: exosomes, mesenchymal stem cells, stem cells, osteoarthritis, cartilage injury. These keywords are used as MeSH headings and free text words. Additional searches for relevant references in the included articles and existing systematic reviews are performed manually. In addition, to maximise the search for relevant articles, further articles were identified by reviewing the references of the selected articles. Specific search strategies for PubMed databases are described in Supplementary Material S1. Furthermore, a list of references for related articles was consulted in order to identify additional studies.

### 2.2 Selection process

The retrieved studies will be imported into Endnote X9.1 for the purpose of removing duplicates. The two reviewers (ZW and ZHH) will conduct independent screening of titles and abstracts in accordance with pre-established inclusion and exclusion criteria. Subsequently, the full text will be subjected to a secondary screening. Two reviewers will cross-check the included studies. In the event that consensus cannot be reached, a third reviewer (YSQ) will be consulted to resolve any differences.

### 2.3 Inclusion and exclusion criteria

Inclusion criteria were developed using the Participants, Intervention, Control, Outcome, and Study design (PICOS) approach and are presented in Table 1. In summary, the criteria were based on randomized controlled trials (RCTS) of preclinical *in vivo* animal studies (using rats exclusively) examining the impact of MSC-exos on OA.

**TABLE 1:** PICOS criteria for inclusion of studies.

Participants	Rat model of osteoarthritis
Intervention	Exosome therapy derived from mesenchymal stem cells
Control	Placebo treatment
Outcome	Primary outcome: OARSI score, Mankin score, ICRS score Secondary outcome: Collagen type II, aggrecan, IL-1β, IL-6, IL-10, MMP13, TNF-α
Study design	Randomized controlled trials

OARSI, osteoarthritis research society international; ICRS, international cartilage repair society; IL, interleukin; MMP13, Matrix Metalloproteinase-13; TNF-α, Tumor Necrosis Factor-α.

Exclusion criteria from the study were: 1) Review or conference abstract. 2) No publication. 3) No English. 4) Publication date does not match. 5) Uncontrolled study. 6) No Exosomes group or control group. 7) Combination studies of EVs/exosomes with other drugs.

### 2.4 Data extraction and data items

All pertinent data are extracted independently by two reviewers (ZW and ZHH) from the textual, tabular, and graphical components of the final qualified article. The data set included the following variables: first author name, year of publication, study design, sample size, subject population, therapeutic intervention method, intervention duration, outcomes, and conclusions. The study did not provide raw data in a format that could be used for analysis, so we contacted the authors to request it. In the event that meta-analysis data, such as mean and standard difference, can only be obtained from charts, the GetData Graph Digitizer 2.26 software employed to extract the requisite data. Should any discrepancy arise between the two examiners, it is resolved through discussion and consensus. In the event that consensus cannot be reached, the matter is referred to a third reviewer (YSQ) for resolution.

### 2.5 Assessments of risk of bias

Two independent reviewers (ZW and ZHH) assessed the risk of bias in the *in vivo* animal studies included in this document. The Systematic Review Centre for Laboratory animal Experimentation (SYRCLE) report on risk of bias assessment in preclinical studies was used to assess the risk of bias in the *in vivo* studies included (Hooijmans et al., 2014), which includes 10 items on six types of bias. Differences between the two reviewers in the two methods are resolved by discussion and consensus.

### 2.6 Data synthesis

GetData Graph Digitizer, Review Manager (RevMan) 5.4.1, Stata IC 15 and Stata 18 were used for systematic review and meta-analysis, data extraction, and processing, respectively. Collection of mean and standard deviation (SD) data. For studies reporting only the mean standard error (SEM), the SEM was converted to SD using the following formula SD = SEM × (√n) (where “n” is the number of animals in the experimental or control group). The mean and SD are used as inputs to the data processing tool to generate the Weighted Mean Difference (WMD) and Standardized Mean Difference (SMD) and their respective 95% confidence intervals (95% CI), with the SMD selected as the final effective measure.

Heterogeneity was assessed using Q statistical tests and I^2^ tests. Heterogeneity between studies was considered if P ≤ 0.05 or I^2^ > 50%. If the heterogeneity assessment results of the two test methods are inconsistent, the I^2^ test results are used because they are more reliable than the Q statistical test. Use subgroup analysis, sensitivity analysis, or other analyses to account for significant heterogeneity among studies.

A Bayesian random effects network meta-analysis of cell type of origin and injection frequency of MSCs-exos was performed using Stata 18 software. A network evidence plot was constructed for each outcome measure, where each node represents an intervention, the line connecting the nodes represents a head-to-head comparison between the two interventions, and the width of the line represents the number of studies comparing the two interventions. In the absence of a closed loop, the consistency model is used for analysis. The effect sizes of each outcome measure are combined by SMD and 95% CI and the probability rankings are plotted.

Publication bias was assessed by constructing a funnel plot. If the funnel plot shows a slight asymmetry, the Egger test is used to verify the authenticity of the asymmetry. P > 0.05 indicates no asymmetry. At the same time, when the sample size is 10 or greatera, trim-and-fill method was used to estimate the impact of publication bias on the results for data with a p-value of 0.05 or less. In addition, a meta-based impact analysis was used as a sensitivity analysis to exclude the effects of small sample sizes to determine the stability of the results. Finally, in meta-analyses, results are considered significant when P < 0.05.

## 3 Result

### 3.1 Study selection

**FIGURE 1:**
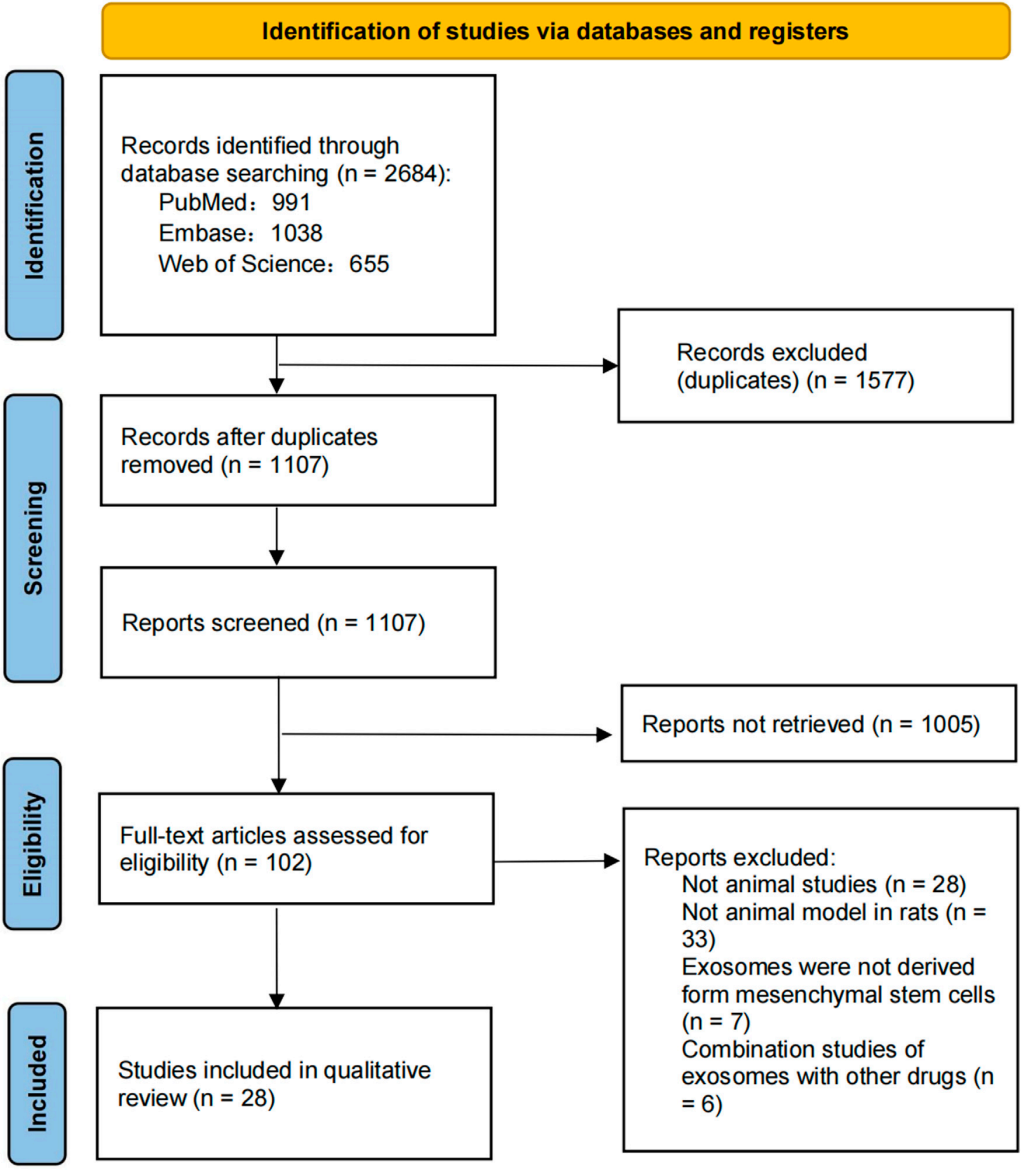
PRISMA flowchart of the systematic literature review.

A total of 2,684 studies were identified through a database search, and the remaining 1,107 studies were identified after duplicate removal. During this period, 1,577 studies were excluded and titles and abstracts were screened according to the above inclusion and exclusion criteria, resulting in 102 full-text articles for relevance assessment. After full-text screening and searching for additional studies in the reference list, 28 studies were selected for qualitative analysis. The selection process and screening flowchart of PRISMA is as follows (Figure 1).

### 3.2 Study characteristics

The 28 studies were conducted in 2017, 2020, 2021, 2022, 2023 and 2024, three experiments were conducted in South Korea, Egypt and Italy, and the remaining 25 experiments were conducted in China. The total sample size was 347 animals, of which 174 received exosomes treatment and the rest received placebo. Characteristics of all 28 studies were collected and listed (Table 2), including (but not limited to) animal models used, exosome sources, extraction methods, injection frequency, therapeutic dose, and evaluation measures.

**TABLE 2:** Characters of included studies in the meta-analysis.

Study	Year	Model	MSCs species	Injection frequency	Injection frequency	Dose	Cartilage quantitative Evaluation	Other evaluation	Main findings	Mechanism
Tao et al.	2017	ACLT and DMM	SMSCs	No refer	Once a week	100 μL 10^11^ particles/mL	OARSI score	None	SMSC-140-Exos prevent OA, promote chondrocyte proliferation and migration, and avoid ECM secretion damage	Wnt5a/Wnt5b activate YAP *via* Wnt signaling; miR-140-5p blocks ECM damage *via* RalA
Woo et al.	2020	MIA	ADSCs	Tangential flow filtration (TFF)	Once a week or twice a week	10^8^ particles were given in a 30 μL volume per joint	Mankin score	IL-1	hASC-EVs promote chondrocyte proliferation, migration, and matrix maintenance under IL-1β stimulation	Suppress M1 macrophage infiltration in the synovium
Zhang et al.	2020	Hulth	BMSCs	Centrifugal ultrafiltration	For 3 days for 4 weeks	50 μL 10^10^ particles/mL	OARSI score	COLⅡ, COLⅩ, IL-1β, TNF-α, IL-6, IL-10, iNOS	BMSC-Exos reduce cartilage damage, inflammation, and promote M2 macrophages in OA.	Polarize macrophages to M2; decrease IL-1β, IL-6, TNF-α, and increase IL-10 levels
He et al.	2020	MIA	BMSCs	Ultracentrifugation	once a week	40µg; 100 µL	OARSI score	COLⅡ, MMP13, ADAMTS5, IL-1β, TNF-α, IL-10, PWT, PWL	BMSC-Exos enhance ECM synthesis, repair cartilage, and alleviate pain in OA rats	Reduce MMP-13, ADAMTS-5, and inflammatory effects of IL-1β
Zavatti et al.	2020	MIA	AFSCs	Centrifugal Filter Units	Every 10 days	100 μg	OARSI score	None	TGFβ-rich AFSC-Exos restore cartilage and modulate macrophage polarization	Promote resolving macrophages; TGFβ content is crucial
Jin et al.	2020	ACLT and DMM	BMSCs	Ultracentrifugation	No refer	No refer	Mankin score	MMP13, IL-1β, TNF-α, IL-6, iNOS	BM-MSC-derived exosomal miR-9-5p reduces inflammation, oxidative stress, and OA-like damage	Targets SDC1 to suppress inflammation and oxidative stress
Pan et al.	2021	collagenase type Ⅱ injection	BMSCs	EVs isolate kit	Once a week	40 μg; 100 μL	OARSI score	None	hMSCs^malat-1^-EVs reduce cartilage degeneration and promote chondrocyte repair	lncRNA malat-1 reduces inflammation and apoptosis in OA chondrocytes
Xu et al.	2021	MIA	BMSCs	ExoQuick-TC™ system	Once a week	140 μg; 100 μL	ICRS score	COLⅡ, SOX9, IL-1β, TNF-α, IL-6, IL-18	BMSC-Exos deliver miR-326 to inhibit pyroptosis and improve OA symptoms	miR-326 targets HDAC3 and STAT1/NF-κB p65 signaling
Jiang et al.	2021	ACLT	BMSCs	Ultracentrifugation	No refer	100 μg; 50 μL	None	COLⅡ, Aggrecan	BMSC-Exos regulate glutamine metabolism to alleviate OA and improve chondrocyte function	Regulate c-MYC to enhance glutamine metabolism
Lu et al.	2021	ACLT and DMM	SMSCs	Ultracentrifugation	once a week	30 μL 10^11^ particles/mL	OARSI score	TNF-α, IL-10	SMSC-EVs carrying miR-26a-5p repair OA cartilage and reduce apoptosis	miR-26a-5p inhibits PTEN, reducing apoptosis and inflammation
Yan et al.	2021	make cartilage defects	hUC-MSCs	Ultracentrifugation	No refer	200 μL; 1 mg/mL	ICRS scores	None	UMSC-Exos with lncRNA H19 promote cartilage repair and suppress senescence	lncRNA H19 acts as ceRNA for miR-29b-3p to upregulate FoxO3
Liao et al.	2021	ACLT and DMM	BMSCs	Ultracentrifugation	Twice a week	100 μL; 400 μg/mL	OARSI score	COLⅡ, Aggrecan, SOX9	LIPUS enhances BMSC-Exos effects on cartilage regeneration and inflammation reduction	Inhibits IL-1β-induced NF-κB activation
Tang et al.	2021	ACLT	hUC-MSCs	Ultrafiltration and Size-exclusion chromatography (SEC)	Once a week	30 μg; 200 μL	OARSI score	COLⅡ, MMP13, ADAMTS5	hUC-MSC-sEVs reduce cartilage damage, promote repair, and maintain homeostasis in OA.	Reduce MMP-13, ADAMTS-5, TNF-α, and increase COL II expression
Lin et al.	2021	MIA	DPSCs	Ultracentrifugation	once a week	50 µL; 5 × 10^10^ particles/mL	OARSI score	None	DPSC-Exos enriched with miR-140-5p promote cartilage repair and prevent OA progression	Regulate apoptosis-related proteins to reduce chondrocyte apoptosis
Xu et al.	2021	DMM	SF-MSCs	Ultracentrifugation	once a week	100µM; 100 μL	OARSI score	None	E7-Exos deliver KGN, improving chondrogenesis and OA therapy	Targeted delivery of KGN enhances SF-MSC differentiation and cartilage repair
Li et al.	2022	ACLT and DMM	UMSCs	Ultracentrifugation	twice a week	100 µL 10^11^ particles/mL	OARSI score	COLⅡ, MMP-13	hUC-MSC-Exos repair cartilage and prevent OA progression	Inhibit inflammatory cytokines; promote macrophage polarization
Xu et al.	2022	ACLT	ADSCs	Ultracentrifugation	Once a week	1 × 10^9^ particles/mL	OARSI score	COLⅡ, Aggreca (no data)	PEMF-exposed AMSC-Exos enhance cartilage regeneration and suppress inflammation	PEMF increases ECM synthesis and reduces IL-1β, MMP-13, and caspase-1 expression
Lou et al.	2023	ACLT and DMM	BMSCs	Ultracentrifugation	No refer	10 μL/week	OARSI score	MMP13, iNOS	F-MSC-Exos reduce ECM degradation, promote autophagy, and protect cartilage	miR-146b-5p inhibits TRAF6 and PI3K/Akt/mTOR signaling
Li et al.	2023	MIA	ADSCs	Ultracentrifugation	No refer	100 µg	OARSI score	COLⅡ	hADSC-Exos mitigate chondrocyte degradation and fibrosis in OA.	miR-376c-3p represses WNT3/WNT9a and Wnt/β-catenin signaling
Kong et al.	2023	DMM	SMSCs	HieffTM Quick Exo-some Isolation Kit	once a week	50 μg, 100 μg or 200 μg; 50 μL	OARSI score	COLⅡ, AggrecanMMP13, Aggrecan, IL-1β, TNF-α, IL-6	SMSC-Exosomal miR-320c repairs cartilage and suppresses ECM degradation in OA.	Targets ADAM19-dependent Wnt signaling to reduce apoptosis and ECM loss
Zhao et al.	2023	ACLT and DMM	ADSCs	Ultracentrifugation	once a week	50 μL 2 × 10^10^ particles/mL	OARSI score	COLⅡ, MMP-13	CAP-MSC-Exos deliver miR-199a-3p, protecting cartilage from OA damage	miR-199a-3p modulates mTOR-autophagy and prevents cartilage degradation
Meng et al.	2023	ACLT	ADSCs	Total exosome isolation reagent	No refer	10 µL 10^10^ particles/mL	OARSI score, Mankin score	COLⅡ	TE-Exos enhance cartilage matrix synthesis and repair in OA.	miR-451-5p modulates chondrocyte activity and ECM synthesis
Sun et al.	2023	MIA	SMSCs	Ultracentrifugation	Every 3 days	100 μL 10^11^ particles/mL	Mankin score	COLⅡ, Aggreca (no data)	BMP-7-Exos promote cartilage regeneration and reduce inflammation in OA.	Induce M2 macrophage polarization; enhance chondrocyte proliferation and migration
Li et al.	2024	ACLT and DMM	BMSCs	Ultracentrifugation	Once a week	100 μg; 50 μL	OARSI score	IL-1β, TNF-α, IL-6, IL-10, iNOS	BMSC-Exos prevent OA by alleviating oxidative stress and cartilage damage	Inhibit PINK1/Parkin signaling and promote M2 macrophage polarization
Yang et al.	2024	ACLT with DMM or collagenase Type II injection	hUC-MSCs	HieffTM Quick Exo-some Isolation Kit	No refer	5 × 10^9^ exosomes injection	None	COLⅠ, COLⅡ	hUC-MSC-Exos improve cartilage regeneration and reduce inflammation	Downregulate MMP-13 and ADAMTS-5, increase COL II expression
Cheng et al.	2024	DMM	BMSCs	Qiagen exosome extraction kit	Twice a week	50 µL	OARSI score	iNOS	BMSC-Exos protect chondrocytes from ferroptosis and OA progression	Disrupt METTL3-m6A-ACSL4 axis to reduce ROS and ferroptosis
El-Din et al.	2024	MIA	BMSCs	Centrifugal ultrafiltration	Once a week	100 µg	OARSI score (no data)	COLⅡ, Aggrecan, IL-1β, TNF-α, IL-10, IL-4, ROS, GSH, MDA	BM-MSC-Exos mitigate OA symptoms and promote cartilage repair	Regulate circYAP1/miRNA-21/TLR7 pathway; increase aggrecan and COL II expression
Dong et al.	2024	DMM	BMSCs	Ultracentrifugation	Twice a week	200 mL	OARSI scores	None	Quercetin-BMSC-Exos prevent chondrocyte apoptosis and OA progression	miR-124-3p targets TRAF6 to block MAPK/p38 and NF-κB signaling

The exosome used in the study was derived from a variety of human or rat/mouse MSCs, including bone marrow mesenchymal stem cells (BMSCs), synovial mesenchymal stem cells, synovial mesenchymal stem cells (SMSCs), umbilical cord mesenchymal stem cells (UMSCs), amniotic fluid mesenchymal stem cells (AFSCs), and adipose tissue-derived mesenchymal stem cells (ADSCs). Although exosomes are derived from cells of different species, the results of each study independently demonstrated their efficacy against OA in animal models. The extraction methods used were tangential flow filtration (TFF), ultrafiltration and size-exclusion chromatography (SEC). Centrifugal ultrafiltration, ultracentrifugation and isolation kit. One experiment did not report the extraction method.

All trials directly compared the exosome treatment group with the placebo group. The route of administration in the study was knee injection, and the frequency of exosome treatment was divided into every 3 days for 4 weeks, every 3 days, every 10 days, weekly, and twice a week. Five trials did not report the frequency of administration. The duration of treatment in all trials ranged from 1 week to 6 months. The results collected included OARSI, Mankin, ICRS, collegen II, aggrecan, IL-1, IL-6, IL-10, MMP13, TNF-α.

### 3.3 Risk of bias in studies

The results of the SYRCLE bias risk assessment in animal studies are shown in Table 3. Across all 28 randomized controlled studies, baseline characteristics were reported to be comparable among experimental groups in the animal models. Four studies (14.3%) explicitly described the method of random sequence generation for animal allocation and were therefore assessed as having a low risk of selection bias. In contrast, the remaining studies merely mentioned the use of “randomization” without detailing the sequence generation process, and were consequently judged as having an unclear risk of bias. Furthermore, five studies (17.9%) reported that outcome assessors were blinded to the intervention, and these were rated as having a low risk of detection bias. However, due to the difficulty of weighting specific items, an overall score is not recommended (Hooijmans et al., 2014), the average number of items in the included studies that met the criteria (selected as “yes”) was 4.4, with moderate results in the range of 4-5, indicating that the methodological quality of the included studies was reliable and acceptable.

**TABLE 3:** SYRCLE Risk of Bias Assessment of animal studies (Included 29 *in vivo* animal studies).

Assessment checklist	Selection bias 1	Selection bias 2	Selection bias 3	Performance bias 1	Performance bias 2	Detection bias 1	Detection bias 2	Attrition bias	Reporting bias	Other potential bias	Over all risk
Tao et al. (2017)	unclear	yes	unclear	unclear	unclear	unclear	yes	yes	yes	yes	5
He et al. (2020)	unclear	yes	unclear	unclear	unclear	unclear	unclear	yes	yes	yes	4
Zhang et al. (2020)	unclear	yes	unclear	unclear	unclear	unclear	unclear	yes	yes	yes	4
He et al. (2020)	unclear	yes	unclear	unclear	unclear	unclear	unclear	yes	yes	yes	4
Zavatti et al. (2020)	unclear	yes	unclear	unclear	unclear	unclear	unclear	yes	yes	yes	4
Jin et al. (2020)	unclear	yes	unclear	unclear	unclear	unclear	unclear	yes	yes	yes	4
Pan et al. (2021)	unclear	yes	unclear	unclear	unclear	unclear	unclear	yes	yes	yes	4
Xu et al. (2021)	unclear	yes	unclear	unclear	unclear	unclear	unclear	yes	yes	yes	4
Jiang et al. (2021)	unclear	yes	unclear	unclear	unclear	unclear	yes	yes	yes	yes	5
Lu et al. (2021)	unclear	yes	unclear	unclear	unclear	unclear	unclear	yes	yes	yes	4
Yan et al. (2021a)	unclear	yes	unclear	unclear	unclear	unclear	yes	yes	yes	yes	5
Liao et al. (2021)	unclear	yes	unclear	unclear	unclear	unclear	unclear	yes	yes	yes	4
Tang et al. (2021)	unclear	yes	unclear	unclear	unclear	unclear	unclear	yes	yes	yes	4
Li et al. (2021)	unclear	yes	unclear	unclear	unclear	yes	unclear	yes	yes	yes	5
Xu et al. (2021)	unclear	yes	unclear	unclear	unclear	unclear	yes	yes	yes	yes	5
Li et al. (2022)	unclear	yes	unclear	unclear	unclear	unclear	yes	yes	yes	yes	5
Xu et al. (2022)	unclear	yes	unclear	unclear	unclear	unclear	unclear	yes	yes	yes	4
Lou et al. (2023)	unclear	yes	unclear	unclear	unclear	unclear	unclear	yes	yes	yes	4
Li et al. (2022)	unclear	yes	unclear	unclear	unclear	yes	unclear	yes	yes	yes	5
Kong et al. (2023)	unclear	yes	unclear	unclear	unclear	unclear	unclear	yes	yes	yes	4
Zhao et al. (2023)	unclear	yes	unclear	unclear	unclear	unclear	yes	yes	yes	yes	5
Meng et al. (2023)	unclear	yes	unclear	unclear	unclear	unclear	unclear	yes	yes	yes	4
Sun et al. (2023)	unclear	yes	unclear	unclear	unclear	unclear	unclear	yes	yes	yes	4
Li et al. (2022)	unclear	yes	unclear	unclear	unclear	unclear	unclear	yes	yes	yes	4
Yang et al. (2024)	unclear	yes	unclear	unclear	unclear	yes	unclear	yes	yes	yes	5
Cheng et al. (2024)	unclear	yes	unclear	unclear	unclear	yes	unclear	yes	yes	yes	5
El-Din et al. (2024)	yes	yes	unclear	unclear	unclear	unclear	unclear	yes	yes	yes	4
Dong et al. (2024)	unclear	yes	unclear	unclear	unclear	unclear	unclear	yes	yes	yes	4

Selection bias 1: Was the allocation sequence adequately generated and applied?, Selection bias 2: Were the groups similar at baseline or were they adjusted for confounders in the analysis?, Selection bias 3: Was the allocation adequately concealed?, Performance bias 1: Were the animals randomly housed during the experiment?, Performance bias 2: Were the caregivers and/or investigators blinded from knowledge which intervention each animal received during the experiment?, Detection bias 1: Were animals selected at random for outcome assessment?, Detection bias 2: Was the outcome assessor blinded?, Attrition bias: Were incomplete outcome data adequately addressed?, Reporting bias: Are reports of the study free of selective outcome reporting?, Other potential bias: Was the study apparently free of other problems that could result in high risk of bias?

### 3.4 Results of syntheses

#### 3.4.1 OARSI score

##### 3.4.1.1 Traditional meta-analysis

**FIGURE 2:**
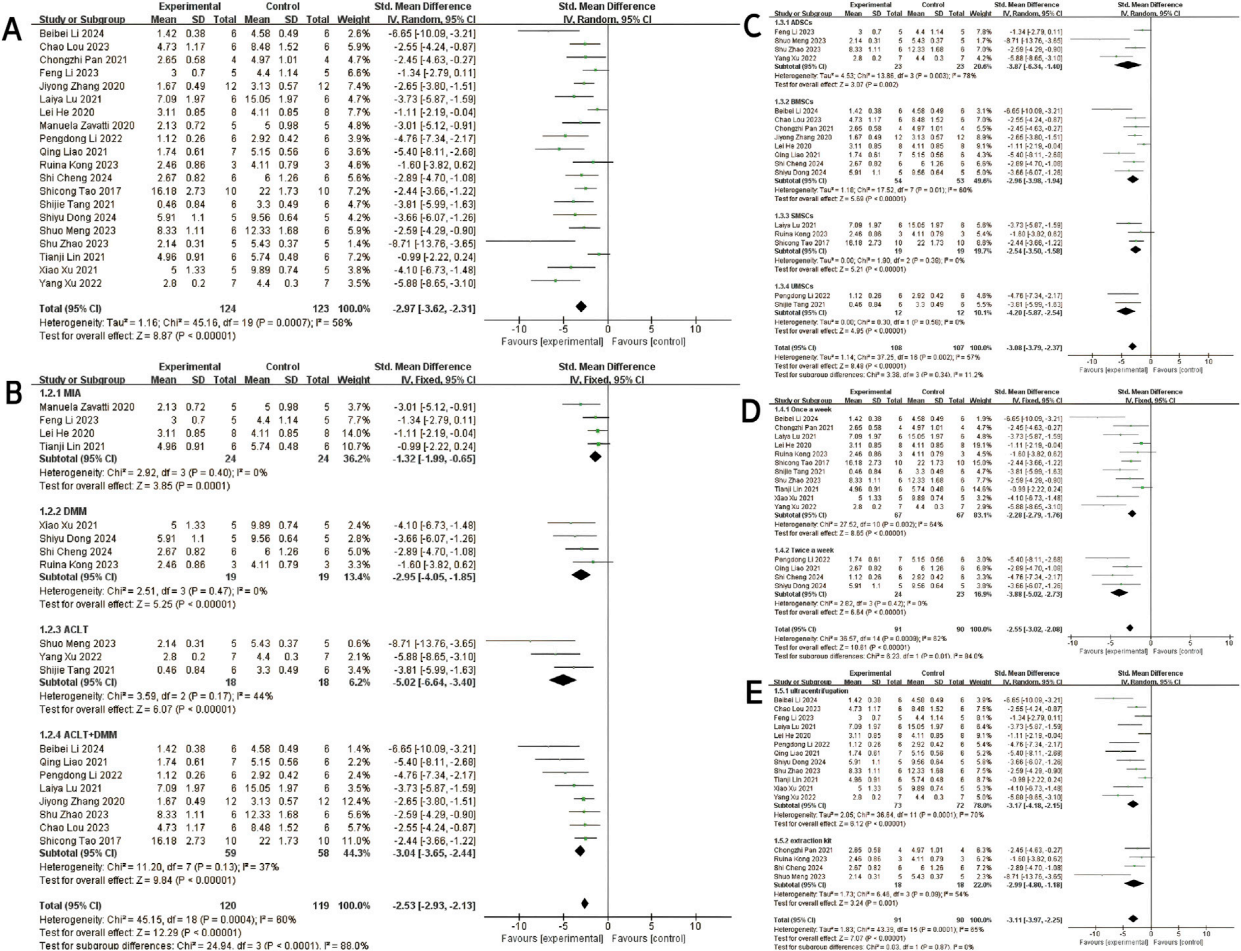
OARSI score: **(A)** Forest plots depicting the comparison between the total experimental and control groups. **(B–E)** Subgroup analysis was performed using the modelling method, MSCs’ type, injection frequency and isolation method.

**FIGURE 3:**
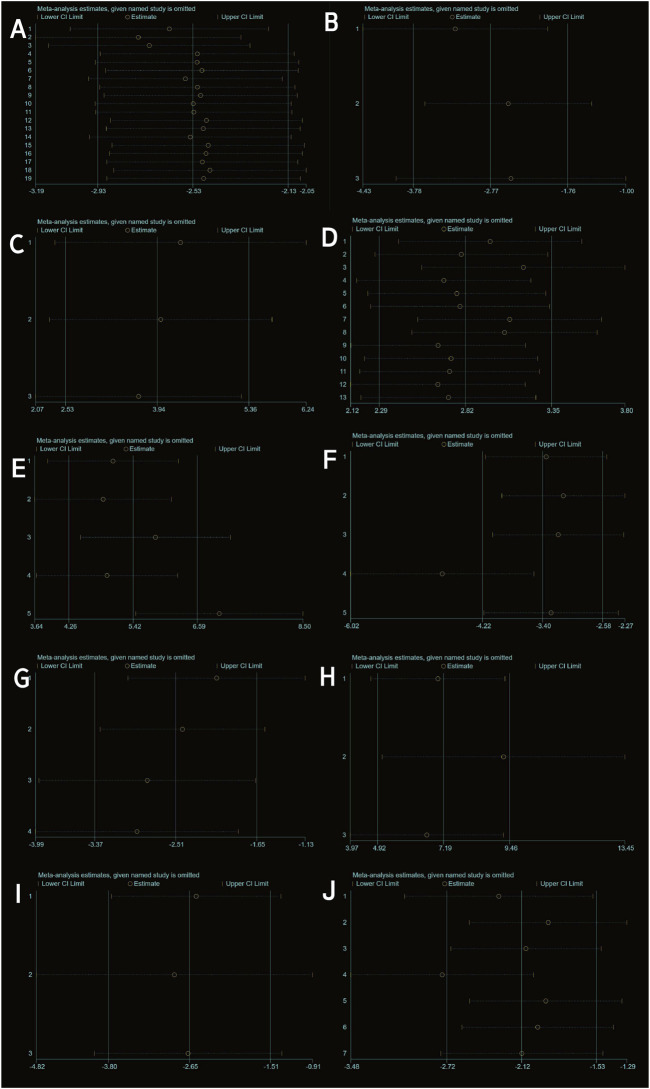
Sensitivity analysis: **(A)** OARSI score. **(B)** Mankin score. **(C)** ICRS score. **(D)** Type II collagen. **(E)** Aggrecan. **(F)** IL-1β. **(G)** IL-6. **(H)** IL-10. **(I)** MMP13. **(J)** TNF-α.

Twenty studies (Pei et al., 2024; Lou et al., 2023; Pan et al., 2021; Gögele et al., 2023; He et al., 2020; Liao C. D. et al., 2021; Zavatti et al., 2020; Zhang et al., 2020; Xu et al., 2021; Kong et al., 2023; Cheng et al., 2024; Dong et al., 2024; Zhao et al., 2023; Lu et al., 2021; Tao et al., 2017; Liao Q. et al., 2021; Li et al., 2022; Meng et al., 2023; Xu et al., 2022; Tang et al., 2021) with a total of 247 subjects reported the OARSI score in their experimental and control groups. The Q test and I^2^ test between studies showed significant heterogeneity (P = 0.0007 < 0.05, I^2^ = 58% > 50%). The results from the random effects model were SMD = −2.97, 95% CI [-3.62, −2.31], P < 0.00001 (Figure 2A.). Therefore, a subgroup analysis was performed to reduce the heterogeneity below 50%. The studies were further divided into three subgroups including MIA、DMM、ACLT、ACLT and DMM according to the different animal models. I^2^ was successfully reduced in each subgroup (MIA: P = 0.40 > 0.05, I^2^ = 0% < 50%; DMM: P = 0.47 > 0.05, I^2^ = 0% < 50%; ACLT: P = 0.17 > 0.05,I^2^ = 44% < 50%; ACLT and DMM: P = 0.13 > 0.05, I^2^ = 37% < 50%). Meanwhile, exosomes therapy increased the OARSI score and reduced the risk of OA cartilage damage according to the SMD results of the four subgroups (MIA: SMD = −1.32, 95% CI [-1.99, −0.65, P < 0.0001; DMM: SMD = −2.95, 95% CI [-4.05,-1.85], P < 0.00001; ACLT: SMD = −5.02, 95% CI [-6.64,-3.40], P < 0.0001; ACLT and DMM: SMD = −3.04, 95% CI [-3.65,-2.44], P < 0.0001) (Figure 2B). The pooled effect size did not change significantly after individual study exclusion in the sensitivity analysis (Figure 3A). The majority of the included studies fell within the 95% confidence interval of the inverted funnel chart, indicating a lower risk of publication bias (Figure 6A). This indicated that the results were relatively robust and reliable.

At the same time, subgroups were established based on the type of MSCs, extraction method and injection frequency. Subgroup analysis based on cell type revealed that MSCs-exos exhibited therapeutic effects on OA, as evidenced by significantly reduced histological scores compared to the negative control group (Total SMD = −3.08, 95% CI [-3.79, −2.37], p = 0.002, I^2^ = 57%) (Figure 2C). Subsequent subgroup analysis based on injection frequency demonstrated that the “once a week” group showed a significant effect with considerable heterogeneity (SMD = −2.28, 95% CI [-2.79, −1.76], p < 0.00001, I^2^ = 84%), whereas the “twice a week” group exhibited a stronger therapeutic effect with no observed heterogeneity (SMD = −3.88, 95% CI [-5.02, −2.73], p < 0.00001, I^2^ = 0%) (Figure 2D). The difference between the two subgroups was statistically significant (p = 0.01), suggesting that injection frequency may influence therapeutic efficacy.

Subgroup analysis based on exosomes isolation method showed that exosomes purified by ultracentrifugation resulted in a significant therapeutic effect (SMD = −3.17, 95% CI [-4.18, −2.15], I^2^ = 70%), albeit with moderate heterogeneity. Similarly, those isolated using commercial extraction kits also demonstrated a significant effect (SMD = −2.99, 95% CI [-4.80, −1.18], I^2^ = 55%) (Figure 2E). The comparison between subgroups yielded no statistically significant difference (p = 0.87), indicating that the method of exosomes isolation did not significantly affect the overall therapeutic outcome.

##### 3.4.1.2 Network meta-analysis

**FIGURE 4:**
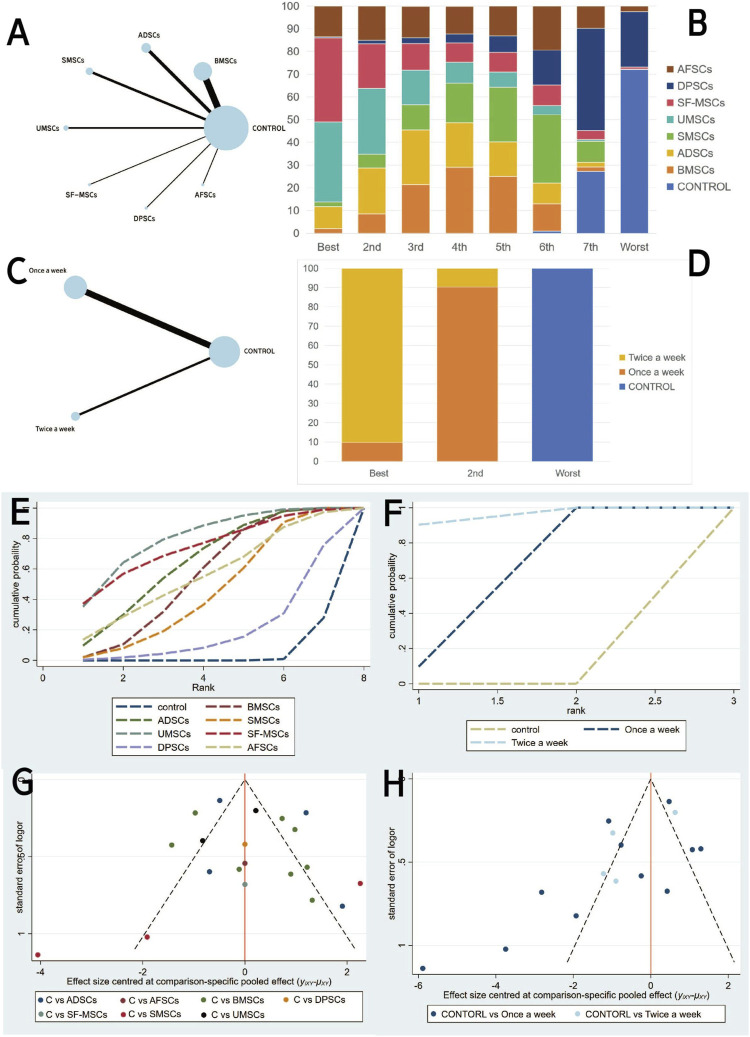
Network meta-analysis of exosomal: **(A,C)** Network evidence maps. **(B,D)** Rank probability ranking plots. **(E,F)** SUCRA cumulative probability plot. **(G,H)** Comparison-adjusted funnel plots. (A,B,E and G为Exosome-derived MSCs types **(C–H)** injection frequencies).

A network meta-analysis was conducted for both cell type and injection frequency. The network plot demonstrated that the majority of stem cell-derived exosome types were directly compared with the control group, with BMSC-exos being the most frequently studied (Figure 4A). The ranking probability plot (Figure 4B) revealed that UMSC-exos and SF-MSC-exos were identified as the most effective, followed by BMSC-exos and ADSC-exos, while DPSC-exos were deemed the least effective.

These results were further corroborated by the SUCRA cumulative probability plot (Figure 4E), in which UMSC-exos and SF-MSC-exos were consistently positioned at the zenith, signifying their superior therapeutic potential in comparison to other stem cell-derived exosomes.

With regard to injection frequency, the network meta analysis (Figure 4C) incorporated direct comparisons between weekly, twice-weekly, and control groups. The ranking probability plot (Figure 4D) demonstrated that the twice-weekly injection group had the highest likelihood of being most effective, followed by the once-weekly injection group, with the control group having the lowest likelihood of efficacy. This finding was corroborated by the SUCRA results (Figure 4F), which demonstrated a pronounced advantage for the twice-weekly regimen.

The comparison-adjusted funnel plots (Figures 4G,H) appeared largely symmetrical, suggesting a low risk of publication bias.

#### 3.4.2 Mankin score

**FIGURE 5:**
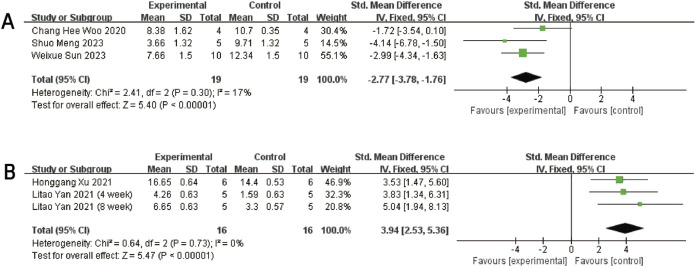
Forest plots depicting the comparison between the experimental and control groups: **(A)** Mankin score. **(B)** ICRS score.

**FIGURE 6:**
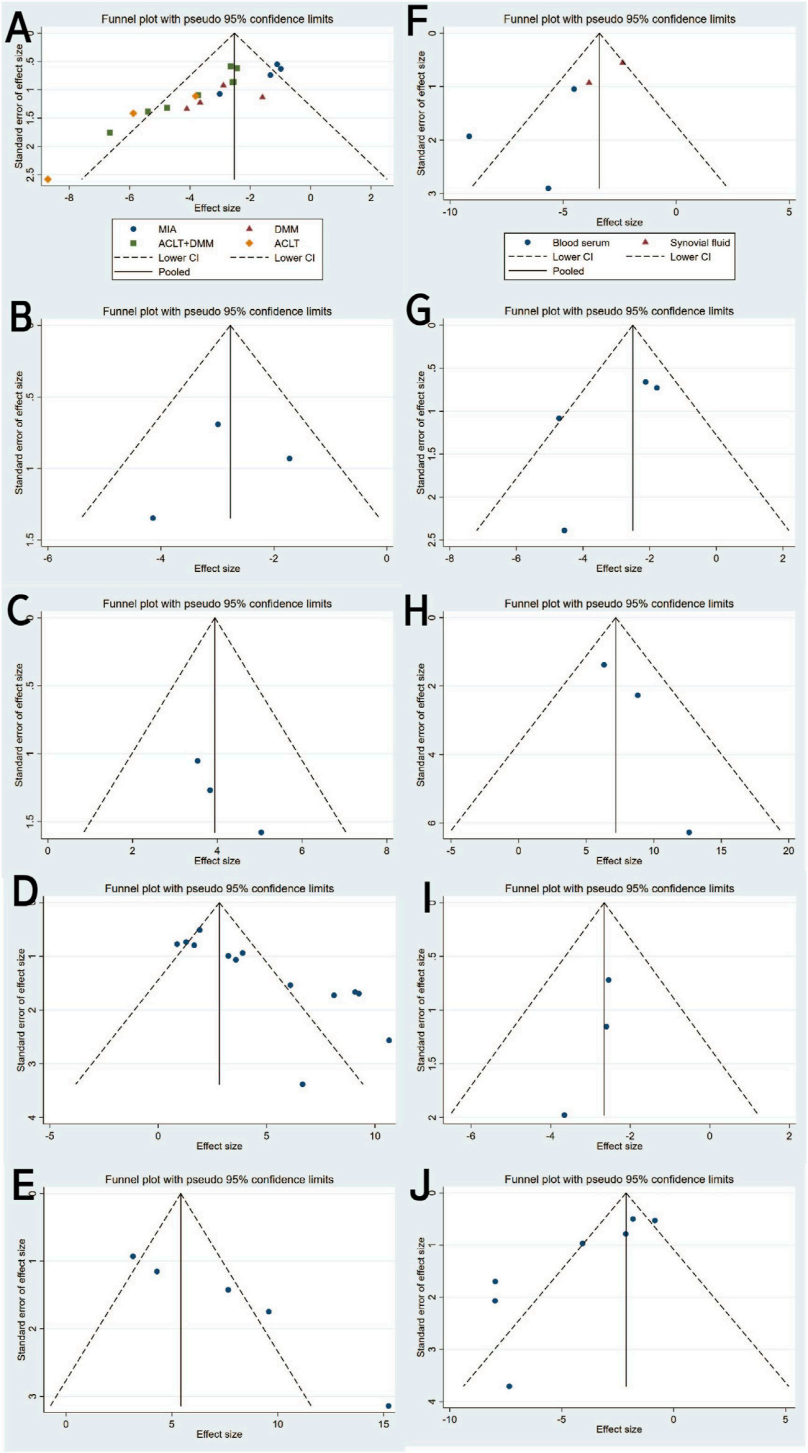
Funnel plot with pseudo-95% confidence limits: **(A)** OARSI score. **(B)** Mankin score. **(C)** ICRS score. **(D)** Type II collagen. **(E)** Aggrecan. **(F)** IL-1β. **(G)** IL-6. **(H)** IL-10. **(I)** MMP13. **(J)** TNF-α.

Three studies (Woo et al., 2020; Meng et al., 2023 and Sun et al., 2023) reported Mankin scores for both the experimental and control groups. Heterogeneity among the included studies was low (I^2^ = 17%, P = 0.30 > 0.05), and thus a fixed-effect model was applied for the meta-analysis. The results demonstrated that exosome treatment significantly reduced the Mankin score compared to the control group [SMD: −2.77, 95% CI (−3.78, −1.76), P < 0.00001] (Figure 5A). All included studies fell within the 95% confidence interval in the inverted funnel plot, indicating a low risk of publication bias (Figure 6B). The robustness of the results was evidenced by the fact that the pooled results remained stable during sensitivity analysis (Figure 3B).

#### 3.4.3 ICRS score

Two studies (three trials) (Xu and Xu, 2021; Yan et al., 2021b) reported ICRS scores for the experimental and control groups. Heterogeneity was found to be negligible in the included literature (I^2^ = 0%, p = 0.73 > 0.05), so a fixed-effect model was used for meta-analysis. The results showed that the ICRS score of exosomes treatment was significantly higher than that of the negative control group [SMD: 3.94, 95% CI (2.52, 5.39), P < 0.0001] (Figure 5B). All of the studies were distributed within the 95% CI range of the inverted funnel plot (Figure 6C). The robustness of the results was evidenced by the fact that the pooled results remained stable during sensitivity analysis (Figure 3C).

#### 3.4.4 Type II collagen

**FIGURE 7:**
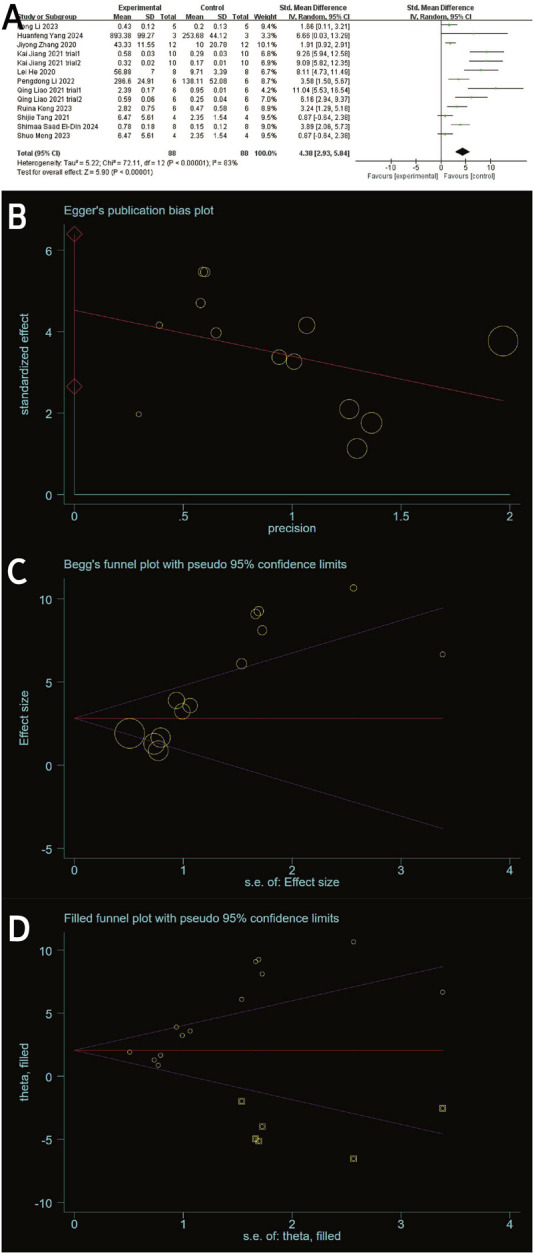
**(A)** Forest plots depicting the comparison between the experimental and control groups. **(B)** Egger’s publication bias plot. **(C)** Begger’s publication bias plot. **(D)** Filled funnel plot with pseudo-95% confidence limits.

Type II collagen data from the experimental and control groups were reported in 11 studies (including 13 trials) (Gögele et al., 2023; Yang et al., 2024; Zhang et al., 2020; He et al., 2020; Li et al., 2022; Kong et al., 2023; Tang et al., 2021; Meng et al., 2023; Jiang et al., 2021; Liao et al., 2021; El-Din et al., 2024) and showed a high degree of heterogeneity (P < 0.00001, I^2^ = 83%), as shown in Figure 7A. Meta-regression was then performed according to animal model, exosome source and time of administration. The meta-regression analysis showed that the variables did not significantly influence the effect size (Supplementary Tables S2–4). Considering the influence of publication bias, the funnel plot shows asymmetry (Figure 6D), and the Egger test results t = 5.32 and P = 0.000 < 0.05 support the asymmetry. Therefore, the stability of the combined effect size was assessed using the pruning and filling method (Figures 7B,C). The results of the fixed effects and random effects models are reported. The random effects model (Q = 71.288, P = 0.000 < 0.05) showed an estimated value (Est) of 4.418 and 95% CI (2.989, 5.847). Six virtual studies were included (Figure 7D) and the data were reanalyzed. The results were Q = 147.477, P = 0.000 < 0.05, and the combined effect size was Est = 2.615 and 95% CI (1.089, 4.141) (Table 4). Despite publication bias, the difference between the two groups was significant (P = 0.000). No contrary situation was observed, showing the reliability of the meta-analysis. In addition, according to the sensitivity analysis, no study data showed a small sample effect, which means that the results of the meta-analysis are reliable (Figure 3D). Analysis of the type II collagen outcome index data using a random effects model showed [SMD: 4.38, 95% CI (2.93, 5.84)], which was considered statistically significant (overall effect test: P < 0.00001). However, due to the high heterogeneity, the results must be interpreted with caution. Nevertheless, all 13 studies showed an increase in type II collagen in the experimental group, suggesting that exosome therapy ameliorated cartilage damage in OA rats.

**TABLE 4:** Process of the trim-and-fill method for Type II collagen (filled meta-analysis).

Method	Pooled Est	95% CI		Asymptotic		No. of studies
		Lower	Upper	z_value	p_value	
Fixed	7.840	4.764	12.904	8.101	0.000	19
Random	8.821	1.838	42.326	2.721	0.007	

Test for heterogeneity: Q = 147.477 on 18 degrees of freedom (p = 0.000).

Moment-based estimate of between studies variance = 9.481.

#### 3.4.5 Aggrecan

**FIGURE 8:**
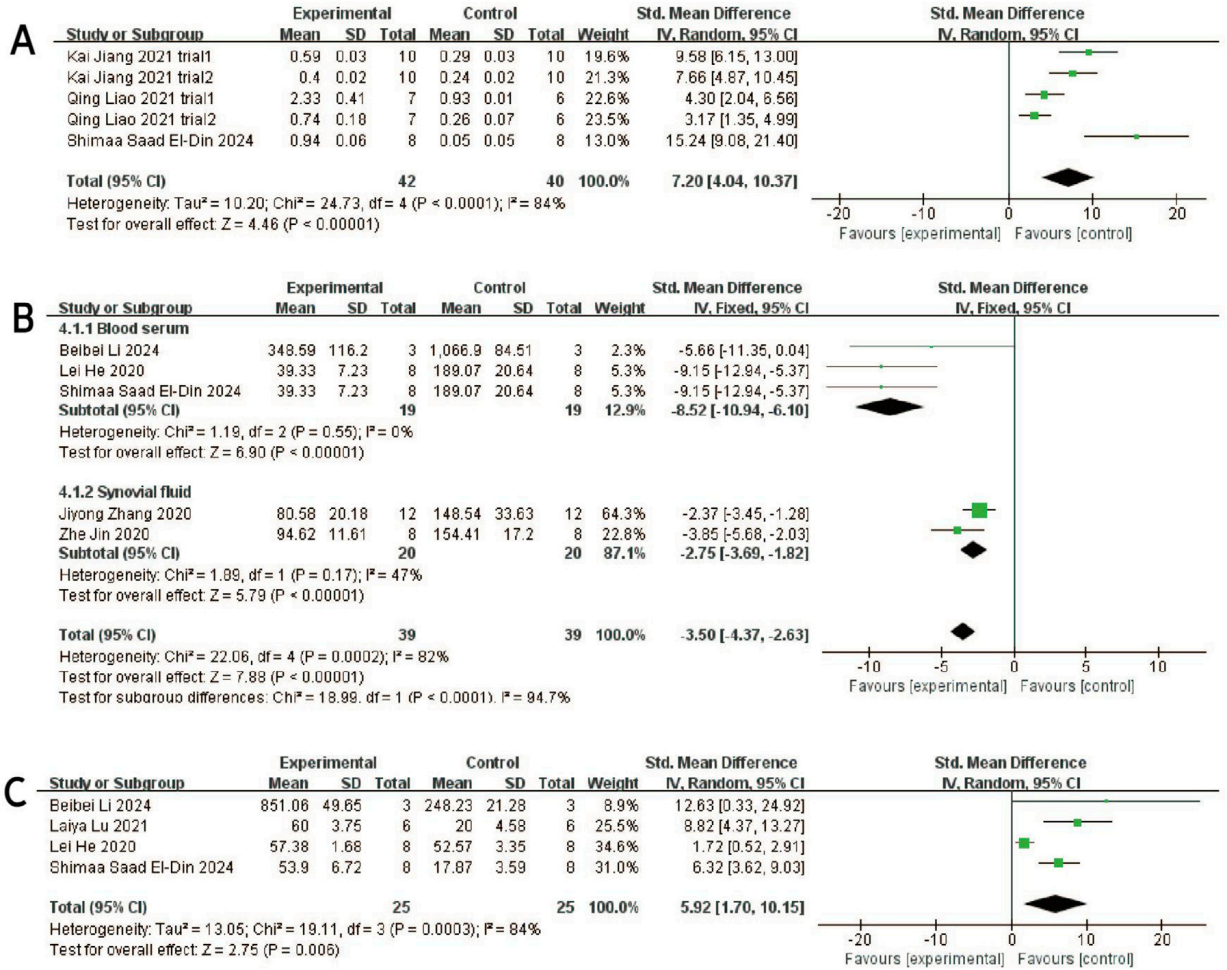
Forest plots depicting the comparison between the experimental and control groups: **(A)** Aggrecan. **(B)** IL-1β. **(C)** IL-6.

Three studies (including five trials) (El-Din et al., 2024; Jiang et al. 2021; Liao et al., 2021) evaluated the effects of exosome treatment on aggrecan expression. Given the substantial heterogeneity among the included studies (I^2^ = 84%, P < 0.0001), a random-effects model was employed to pool the data. To evaluate the robustness of the findings, a sensitivity analysis was performed by sequentially omitting each study. The pooled effect size remained stable throughout the analysis, suggesting that the overall results were not disproportionately influenced by any single study (Figure 3E). Publication bias was assessed using a comparison-adjusted funnel plot (Figure 6E). Although minor asymmetry was observed, most studies were located within the 95% confidence region, indicating a relatively low risk of publication bias. As illustrated in Figure 8A, the meta-analysis demonstrated a statistically significant improvement in the experimental group compared to the control group [SMD = 7.20, 95% CI (4.04, 10.37), P < 0.00001], indicating a favorable effect of exosomes treatment on OA outcomes.

#### 3.4.6 IL-1β

Seven studies (Pei et al., 2024; El-Din et al., 2024; He et al., 2020; Zhang et al., 2020; Z; Jin, Ren, and Qi, 2020; Kong et al., 2023; Woo et al., 2020) examined the effects of mesenchymal stem cell-derived exosomes on IL-1β levels, three of which investigated its serum levels, two investigated its synovial fluid level, another two research investigated its cartilage and synovial level. Studies of IL-1 levels in synovial and cartilage tissues were not included in the meta-analysis due to differences in measurement methods (ELISA and Immunohistochemistry). Sensitivity analysis indicated robust and stable results, with no single study exerting a disproportionate influence (Figure 3F). The funnel plot appeared relatively symmetrical, implying a low risk of publication bias (Figure 6F). A negligible heterogeneity was found among the included literature (I^2^ = 0%, P = 0.55 > 0.05; I^2^ = 47%, P = 0.17 > 0.05), thus a fixed-effects model was used for the meta-analysis (Figure 8B). The results suggested that IL-1β levels in the serum and synovial fluid [SMD: 8.52, 95% CI (−10.94, −6.10), P < 0.01; SMD: 2.75, 95% CI (−3.69, −1.82), P < 0.01] were significantly lower than the negative control group.

#### 3.4.7 IL-6

Four studies (Zhang et al., 2020; Pei et al., 2024; He et al., 2020; Kong et al., 2023) revealed a significant reduction in the outcome measure in the experimental group compared to the control group [SMD = −2.80, 95% CI (−4.18, −1.41), P < 0.0001], as shown in the forest plot (Figure 8C). Although moderate heterogeneity was observed (I^2^ = 52%, P = 0.10), all included studies consistently favored the experimental group, with effect sizes ranging from −0.43 to −4.72, indicating a robust treatment effect. Sensitivity analysis demonstrated the robustness and reliability of the results (Figure 3G). The funnel plot exhibited a relatively symmetrical distribution, with most studies located within the 95% confidence boundaries, suggesting a low risk of significant publication bias (Figure 6G).

#### 3.4.8 IL-10

**FIGURE 9:**
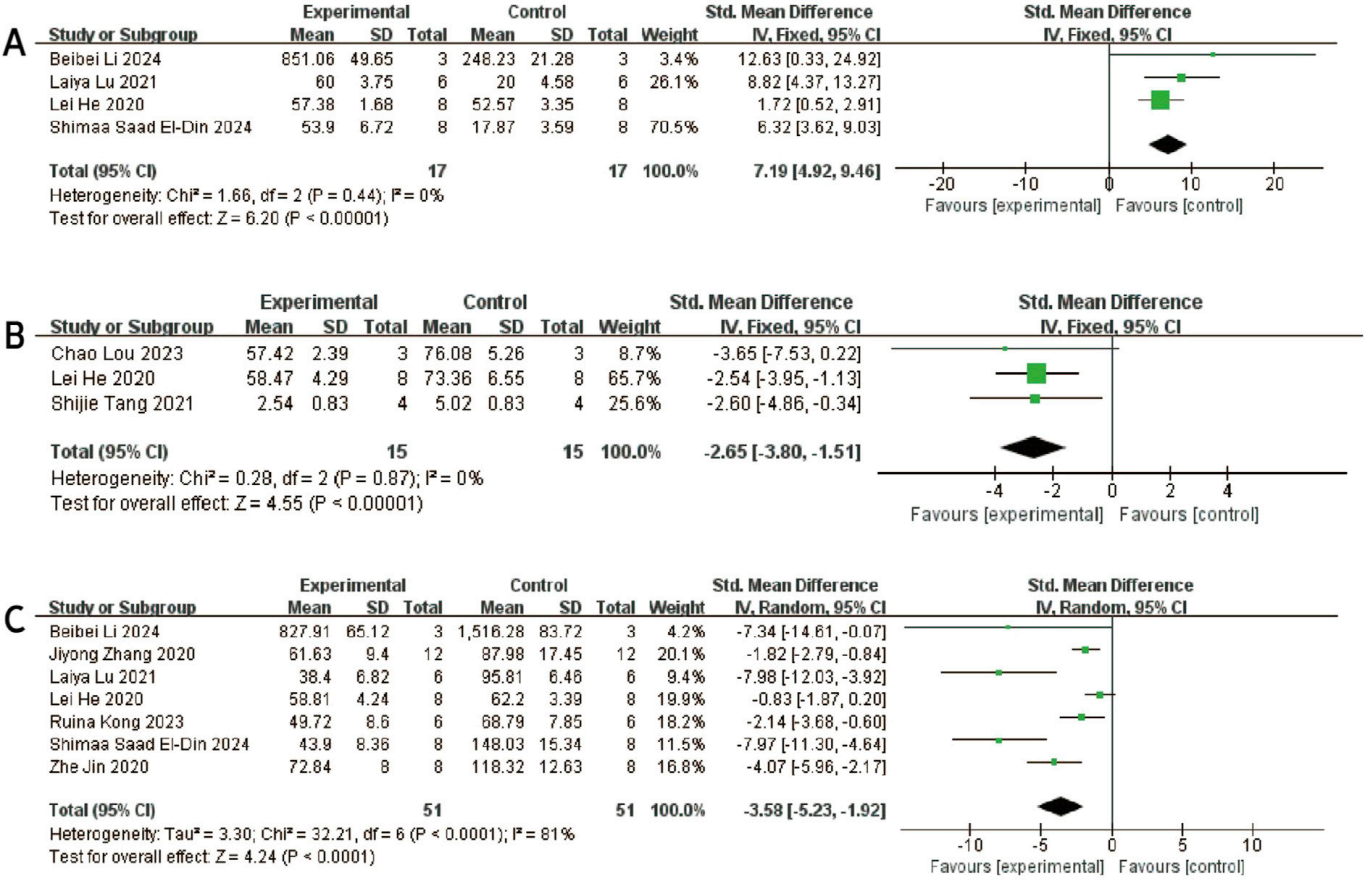
Forest plots depicting the comparison between the experimental and control groups: **(A)** IL-10. **(B)** MMP13. **(C)** TNF-α.

Three studies (Li et al., 2022; El-Din et al., 2024; Lu et al., 2021) reported differences in IL-10 levels between experimental and control groups. Heterogeneity was negligible (I^2^ = 0%, p = 0.436), and therefore a fixed-effects model was applied (Figure 9A). The pooled analysis demonstrated significantly higher IL-10 levels in the experimental group compared to the control group [SMD: 7.19, 95% CI (4.92, 9.46), p < 0.0001]. Sensitivity analysis confirmed the robustness of the findings (Figure 3H), and all studies were distributed within the 95% confidence region in the funnel plot, indicating a low risk of publication bias (Figure 6H). These results suggest that exosome treatment may significantly promote IL-10 secretion.

#### 3.4.9 MMP-13

Three studies (Lou et al., 2023; He et al., 2020; Tang et al., 2021) reported differences in MMP-13 levels between experimental and control groups. Heterogeneity was found to be negligible in the included literature (I^2^ = 0%, p = 0.87 > 0.05), so a fixed-effect model was used for meta-analysis (Figure 9B). The results showed that MMP-13 levels were lower after exosomes treatment than in the negative control group [SMD: 2.65, 95% CI (−3.80, −1.15), P < 0.0001]. All of the studies were distributed within the 95% CI range of the inverted funnel plot (Figure 6I), and sensitivity analysis also proved the robustness of the results (Figure 3I).

#### 3.4.10 TNF-α

Seven studies (Zhang et al., 2020; Jin, Ren, and Qi, 2020; Pei et al., 2024; He et al., 2020; El-Din et al., 2024; Lu et al., 2021; Kong et al., 2023) reported TNF-α data in experimental and control groups, showing high heterogeneity (P < 0.00001, I^2^ = 81%), as shown in Figure 9C. Meta-regression analyses were conducted based on animal model and exosome source. However, the results indicated that these variables did not significantly influence the overall effect size (Supplementary Tables S5 and S6). To assess the risk of publication bias, a funnel plot was generated and revealed marked asymmetry (Figure 6J), suggesting potential bias. Sensitivity analysis further indicated that certain studies may have contributed to instability in the pooled results (Figure 3J). Due to the limited number of studies (n < 10), the trim-and-fill method was not applied, as it could lead to false-positive findings. Despite the substantial heterogeneity observed, the use of exosomes consistently resulted in a significant reduction in TNF-α levels compared to controls, supporting their therapeutic potential.

## 4 Discussion

Over the past years, exosomes derived from MSCs have been shown to have potential as ‘cell-free therapies’ for OA (Ni et al., 2020; Yu, Huang, and Yang, 2022; Wen et al., 2024). In this meta-analysis, pre-clinical studies on the potential of MSCs-derived exosomes for the treatment of OA in animal models were systematically evaluated, including a total of 28 studies. To minimise the differences between animal models, only the rat model of OA was included in this study. Compared with mice, the thickness of cartilage in rats is more similar to that in humans, and research into its gene sequence is more advanced. Quality assessments using the Cycle Bias risk assessment tool showed that most of the studies were of moderate quality. The results show that by delivering miRNAs, proteins and other functional molecules that mediate cartilage repair, inflammation regulation and cell protection, MSCs-derived exosomes significantly improve OA healing.

Cartilage Score is the key index for assessing cartilage repair (Gerwin et al., 2010; Abedian et al., 2013), it is noteworthy that all 27 studies using cartilage scores (OARSI score, Mankin score and ICRS score) showed a significant improvement in OA with MSCs-exos, although the moderate study quality and the inherent variability of animal studies such as OA model creation methods, exosomes dosing and dosing frequency must be taken into account. The meta-analysis of the OARSI score showed high heterogeneity (I^2^ = 58). The results of the subgroup analysis showed that subgroup analysis with different modelling methods showed differences between groups, which was identified as one of the sources of high heterogeneity. Subgroup analysis of exosomes isolation and extraction showed no significant difference between the two methods of ultracentrifugation and kit extraction.

Network meta-analysis demonstrated that UMSC-exos and SF-MSC-exos were associated with more significant treatment outcomes. The beneficial effect of SF-MSC-exos may be attributable to their content of signalling molecules associated with joint tissue and cartilage metabolism, thereby enabling more effective engagement with the local microenvironment (Neybecker et al., 2020). Furthermore, these exosomes are more readily taken up by target cells, such as kondrocytes and synoviocytes, and can effectively regulate the inflammatory, apoptotic and repair responses of these cells. The miRNAs, lncRNA and proteins carried by these exosome-rich fractions from the synovial fluid of OA patients reflect the local pathological state, which in turn may more precisely affect the key signalling pathways of OA (such as NF-κB, Wnt/β-catenin, MAPK, *etc.*) to achieve “self-regulation”. Furthermore, the anti-inflammatory, anti-apoptotic and pro-regenerative bioactive molecules present in UMSC-exos, such as miR-140-5p, TGF-β, IL-10, HGF, are able to effectively inhibit cartilage degeneration and promote matrix formation, regulate macrophage polarisation and T-cell activity, and inhibit the chronic inflammatory process in OA. Furthermore, they have been shown to reduce synovial inflammation and cartilage destruction. In comparison to ADSC-exos or BMSC-exos, they demonstrate enhanced adaptability to the microenvironment of OA lesions, which may underpin their superior repair efficacy. Network analysis of injection frequency demonstrated that twice-weekly injection of exosome was more efficacious in the repair of OA than those injected once weekly, suggesting that the repair of cartilage damage by exosomes may be dose-dependent.

The therapeutic effect of exosomes therapy is primarily achieved through the expression of various non-coding Rnas, including but not limited to miRNAs, lncRNAs, and circRNAs (Headland et al., 2015; Young et al., 2022). In recent years, there has been a gradual increase in the number of researchers focusing on the study of non-coding RNAs of exosomes. The observed differences in therapeutic efficacy among exosomes derived from various MSCs sources may be attributed, at least in part, to variations in their molecular cargo, particularly miRNA composition and proteomic profiles. miRNAs are key regulators of gene expression and play critical roles in modulating inflammation, apoptosis, and tissue regeneration (Chen et al., 2024; Zhang et al., 2023; A, T, and Wl 2024). Distinct exosomal miRNA signatures have been identified across MSCs of different origins, which may influence their downstream biological effects in osteoarthritic joints (Chen, Tan, et al., 2024). For instance, UMSC-exos are often enriched in regenerative and anti-inflammatory miRNAs such as miR-100-5p, miR-1208, and lncRNA H19 (X. Li et al., 2021; Yan et al., 2021a; Yan et al., 2021b; Zhou et al., 2022), whereas exosomes from other sources may lack such potent regulatory molecules or contain miRNAs with divergent functional roles.

In addition to miRNA content, differences in the exosomal proteome likely contribute to the heterogeneity in therapeutic outcomes. Proteomic analyses have revealed that exosomes carry a diverse array of bioactive proteins, including cytokines, growth factors, and extracellular matrix components, which are differentially expressed depending on the cellular origin and microenvironmental conditions (Wang et al., 2020). A total of 382 proteins have been identified as being specific to UMSC-exos. These proteins are involved in the processes of extracellular matrix tissue and extracellular structural tissue, target cell membranes to promote wound healing and angiogenesis in mice, and promote proliferation, migration, and angiogenesis of UMSCs(Zhang et al., 2022). These proteins can interact with recipient cells and modulate signaling pathways involved in cartilage repair, immune regulation, and angiogenesis (Yang et al., 2023). Therefore, the source-dependent variability in both miRNA and protein composition may account for the differential capacity of exosomes to modulate the osteoarthritic microenvironment and promote joint tissue regeneration.

Type II collagen is the most abundant collagen in articular cartilage, accounting for over 90% of the collagen in the cartilage matrix (R. Xu et al., 2023; Henrotin et al., 2007; Poole et al., 2002). It interacts with proteoglycan and other matrix components to form a stable network structure that supports chondrocytes and dissipates mechanical stress (Nham et al., 2019). MSCs-exos can effectively repair damaged cartilage by enhancing the proliferation and migration of chondrocytes and upregulating the expression of type II collagen and aggrecan (Tao et al., 2017; Huang et al., 2021). MMP-13 and ADAMTS-5 are the main enzymes that degrade type II collagen and aggrecan, respectively. Exosomes play a certain protective role in articular cartilage by reducing the expression of related degradative enzymes. hUC-MSCs SEVs not only reduced the levels of MMP-13 and ADAMTS-5, but also maintained cartilage homeostasis by upregulating type II collagen (Tang et al., 2021). MSCs-exos, including those derived from SMSCs, DPSCs, and CAP-MSCs, have demonstrated the ability to activate protective signaling pathways (e.g., Wnt5a/Wnt5b-YAP, mTOR-autophagy) to prevent ECM degradation and chondrocyte apoptosis (Tao et al., 2017; Zhao et al., 2023; Liao C. D. et al., 2021).

Apart from promoting cartilage repair, MSC-derived exosomes contribute to immune modulation by regulating cytokine secretion and influencing macrophage phenotype transitions (Wang et al., 2023; Wei et al., 2024). Several studies confirm that exosomes reduce pro-inflammatory cytokines (IL-1β, IL-6, TNF-α) while enhancing anti-inflammatory markers such as IL-10 (Mianehsaz et al., 2019; Kim, Choi, and Kim, 2020; Zhang et al., 2020). This effect is partly mediated by miRNAs like miR-146B-5P and miR-9-5p, which inhibit inflammatory signaling (e.g., TRAF6, PI3K/Akt/mTOR) and reduce oxidative stress (Lou et al., 2023; Jin, Ren, and Qi, 2020). Additionally, TGF-β and BMP-7 enriched exosomes further promote M2-type macrophage polarization and cartilage regeneration (Zavatti et al., 2020; Sun et al., 2023).

The anti-apoptotic and antioxidative capacities of MSCs-exos also contribute to their protective role in OA. Exosomal delivery of miR-124-3p (from quercetin-modified BMSCs) inhibits chondrocyte apoptosis by targeting MAPK/p38 and NF-κB pathways (Dong et al., 2024). Moreover, BMSC-exos can alleviate oxidative stress by regulating PINK1/Parkin signaling and reducing ROS production and ferroptosis *via* the METTL3-m6A-ACSL4 axis (Pei et al., 2024; Cheng et al., 2024).

This systematic review focuses on studies published between January 2017 and July 2024, reflecting the significant interest and research focus on exosomes in the field of cartilage protection and the treatment of OA. The current preclinical evidence tentatively suggests that exosomes may have some efficacy and application potential in the treatment of OA. However, these conclusions are mainly based on animal model studies and the reliability of their extrapolation to the clinic needs to be further verified. Therefore, high-quality *in vivo* studies and randomised controlled clinical trials are essential to fully evaluate the efficacy and safety of exosome therapy.

There are some limitations of the studies in this review, mainly reflected in the differences in exosome types, doses and injection frequencies in the studies, resulting in increased heterogeneity of results. These factors limit the comparability and generalisability of the research findings. The majority of studies included in this meta-analysis were conducted in China, which may introduce potential regional bias and limit the external validity and generalizability of the findings. Therefore, the results should be interpreted with caution. Further high-quality studies from diverse geographic regions are warranted to validate and complement the conclusions of this study. Nevertheless, these preclinical studies provide preliminary evidence that could help guide future well-designed clinical trials and suggest the potential, yet still exploratory, clinical applicability of exosomes in OA treatment.

## 5 Conclusion

Based on 28 studies in animal, exosomes derived from MSCs exhibit considerable potential of alleviating cartilage damage in rats, in which the resarches in BMSCs-exos is particularly extensive. The extant research is limited to qualitative assessments of the therapeutic efficacy of Exosomes, thus it is essential to quantify presion in concentration, dosage and injection frequency. Network meta-analysis further demonstrated that UMSC-exos and SF-MSC-exos were associated with more significant treatment outcomes in OA. Furthermore, network analysis of injection frequency suggested that a twice-weekly regimen may be more suitable for therapeutic use. Despite its limitations, the preliminary evidence is encouraging and suggests that exosomes therapy may represent a promising adjunct in the treatment of OA, warranting further investigation in future clinical trials.

## Data Availability

The raw data supporting the conclusions of this article will be made available by the authors, without undue reservation.
