# Supplementary material for: Mesenchymal stem cell-derived exosomes for the treatment of knee osteoarthritis: a systematic review and meta-analysis based on rat model
Source: Front Pharmacol. 2025 Jun 2;16:1588841. doi: 10.3389/fphar.2025.1588841 (PMC12171193; doi:10.3389/fphar.2025.1588841)
Supplement: Supplementary file 1 [file Supplementaryfile1.docx]

**Table S1. PubMed search strategy.**

| **# No** | **Searches** |
| --- | --- |
| **1** | “Mesenchymal Stem Cells”[Mesh] |
| **2** | “Mesenchymal Stem Cells”[Title/Abstract] OR “Stem Cell, Mesenchymal”[Title/Abstract] OR “Mesenchymal Stem Cell”[Title/Abstract] OR “Stem Cells, Mesenchymal”[Title/Abstract] OR “Mesenchymal Stromal Cells”[Title/Abstract] OR “Mesenchymal Stromal Cell”[Title/Abstract] OR “Stromal Cell, Mesenchymal”[Title/Abstract] OR “Stromal Cells, Mesenchymal”[Title/Abstract] |
| **3** | 1 OR 2 |
| **4** | “Exosomes”[Mesh] |
| **5** | “Exosomes”[Title/Abstract] OR “Secretory Vesicles”[Title/Abstract] OR “Cell-Derived Microparticles”[Title/Abstract] OR “Exosome Multienzyme Ribonuclease Complex”[Title/Abstract] OR “Exo”[Title/Abstract] |
| **6** | 4 OR 5 |
| **7** | “Osteoarthritis”[Mesh] |
| **8** | “Osteoarthritis”[Title/Abstract] OR “Osteoarthritides”[Title/Abstract] OR “Arthritis, Degenerative”[Title/Abstract] OR “Arthritides, Degenerative”[Title/Abstract] OR “Degenerative Arthritides”[Title/Abstract] OR “Degenerative Arthritis”[Title/Abstract] OR “Osteoarthrosis”[Title/Abstract] OR “Osteoarthroses”[Title/Abstract] OR “Osteoarthrosis Deformans”[Title/Abstract] OR “Osteoarthritis, Knee”[Title/Abstract] OR “OA”[Title/Abstract]OR “KOA”[Title/Abstract] |
| **9** | 7 OR 8 |
| **10** | 3 AND 6 AND 9 |

**Table S2**

Meta-Regression Results of Modeling Method on Effect Size (n=13)

| **Variable** | **exp(b)** | **Std. Error** | **t-value** | **P-value** | **95% CI Lower** |
| --- | --- | --- | --- | --- | --- |
| Modeling method | 1.0008 | 0.8196 | 0.00 | 0.999 | [0.1650, 6.069299] |
| Cons | 106.2451 | 239.0192 | 2.07 | 0.062 | [0.7514, 15022.35] |

Note. τ² = 9.761 (between-study variance), I² = 84.38% (residual heterogeneity), Adjusted R² = -12.71%. Analyses performed using REML estimation with Knapp-Hartung adjustment.

**Table S3**

Meta-Regression Results of Injection Frequency on Effect Size (n=13)

| **Variable** | **exp(b)** | **Std. Error** | **t-value** | **P-value** | **95% CI Lower** |
| --- | --- | --- | --- | --- | --- |
| Injection frequency | 2.006635 | 2.432722 | 0.57 | 0.577 | [0.1391946, 28.92772] |
| Cons | 61.77473 | 81.59174 | 3.12 | 0.010 | [3.375166, 1130.646] |

Note. τ² = 9.3 (between-study variance), I² = 82.91% (residual heterogeneity), Adjusted R² = -7.38%. Analyses performed using REML estimation with Knapp-Hartung adjustment.

**Table S4**

Meta-Regression Results of MSCs Types on Effect Size (n=13)

| **Variable** | **exp(b)** | **Std. Error** | **t-value** | **P-value** | **95% CI Lower** |
| --- | --- | --- | --- | --- | --- |
| MSCs types | 0.2253157 | 0.1828619 | -1.84 | 0.093 | [0.0377591, 1.344503] |
| Cons | 1541.704 | 2669.43 | 4.24 | 0.001 | [34.11311, 69675.59] |

Note. τ² = 13 (between-study variance), I² = 83.43% (residual heterogeneity), Adjusted R² = 18.13%. Analyses performed using REML estimation with Knapp-Hartung adjustment.

**Table S5**

Meta-Regression Results of MSCs Types on Effect Size (n=7)

| **Variable** | **exp(b)** | **Std. Error** | **t-value** | **P-value** | **95% CI Lower** |
| --- | --- | --- | --- | --- | --- |
| MSCs types | 0.4528051 | 1.251121 | -0.29 | 0.786 | [0.0003726, 550.2307] |
| Cons | 0.0500495 | 0.1886721 | -0.79 | 0.0463 | [3.10e-06,808.8438] |

Note. τ² = 8.729 (between-study variance), I² = 83.85% (residual heterogeneity), Adjusted R² = -26.84%. Analyses performed using REML estimation with Knapp-Hartung adjustment.

**Table S6**

Meta-Regression Results of Modeling Method on Effect Size (n=7)

| **Variable** | **exp(b)** | **Std. Error** | **t-value** | **P-value** | **95% CI Lower** |
| --- | --- | --- | --- | --- | --- |
| Modeling method | 3.117893 | 5.067355 | 0.70 | 0.515 | [0.0478024, 203.3633] |
| Cons | 0.0028418 | 0.0083492 | -2.00 | 0.103 | [1.49e-06, 5.414638] |

Note. τ² = 8 .273 (between-study variance), I² = 83.91% (residual heterogeneity), Adjusted R² = -20.22%. Analyses performed using REML estimation with Knapp-Hartung adjustment.
